# The Differential Diagnosis of Vertigo

**Published:** 1910-04-30

**Authors:** 


### SPECIAL ARTICLE.

### THE DIFFERENTIAL DIAGNOSIS OF VERTIGO.

Vertigo literally means a "turning" or giddiness. It is a condition which is characterised by a feeling of swimming in the head, oscillation of the body, and rotation of the external objects, sensations which may be associated with a tendency to reel or stagger. It is a disorder of the functions by which the equilibrium of the body is maintained. There are two forms—(*a*) subjective, and (*b*) objective. In the former there is an apparent movement of the patient himself, in the latter there is an apparent movement of external objects.

There are many sensations that may be associated with vertigo, such as a tendency to incline to the right or left, to turn to the right or the left, to fall backwards or forwards, a feeling of sinking downwards or of falling from a height, etc. It is a most distressing symptom, which may be due to many causes, some trifling, some serious, and so requires very careful investigation in order that an accurate diagnosis may be made.

The following are the chief varieties and causes: (1) Aural or labyrinthic—Menière's disease; (2) Organic disease of the brain (*a*) cerebellar tumour, (*b*) cerebral tumour, (*c*) cerebral hæmorrhage; (3) Epileptic; (4) Nervous; (5) Migrainous; (6) Ocular (*a*) due to oculo-motor paralysis, (*b*) due to errors of refraction, (*c*) due to muscular asthenopia; (7) Cardio-vascular (*a*) due to aortic disease, (*b*) due to arterio-sclerosis, etc.; (8) Gastric; (9) Nasal; (10) Laryngeal; (11) Toxic, *e.g.* due to quinine, alcohol, etc.; (12) Nocturnal; (13) Epidemic paralytic (Gerlier's disease).

### Aural or Labyrinthic Vertigo—Meniere's Disease.

This form of vertigo usually occurs in severe paroxysms, and is associated with tinnitus and deafness. It rarely affects people under 30 years of age, and it is commoner in men than in women. It is due to disease of the labyrinth and the nerve-endings which it contains. The chief causes are:—

1. Inflammatory disease of the labyrinth—rheumatic, gouty, syphilitic.
2. Hæmorrhage into the labyrinth—*e.g.* in leuchæmia.
3. Degenerative change in the membranes (senile).
4. Organic disease of the vestibular part of the auditory nerve.
5. Increased labyrinthine pressure, which may be due to:—
   (*a*) Otitis media.
   (*b*) Cerumen blocking the external auditory meatus.
   (*c*) Violent syringing of the ears.
   (*d*) Obstruction of the eustachian tube.
   (*e*) Spasm of the tensor tympani muscle.
   (*f*) Paralysis of the stapedius.

The attacks are characterised by the sudden onset of vertigo, which may occur spontaneously or be excited by some sudden movement such as coughing or sneezing. The paroxysm may be so severe that the patient falls to the ground, and for a moment there may be a loss of consciousness. There may be only a tendency to fall or turn, or there may be a sensation as if external objects are moving; or both these sensations may be associated. Tinnitus and deafness are also present in the majority of cases. The deafness varies in intensity; it may be bilateral or unilateral, generally the former.

After the vertigo has lasted for a short time there is usually a feeling of nausea, which is followed by vomiting, and this is succeeded by pallor and the occurrence of a cold, clammy sweat which breaks out on the skin. In some cases slight nystagmus and diplopia may be noticed during the paroxysm. The diagnosis of aural vertigo, therefore, is based on the association of paroxysms of vertigo with tinnitus and deafness, due to morbid changes disturbing the functions of the labyrinth.

### Organic Disease of the Brain.

*Cerebellar Tumour.*—Vertigo is one of the most constant symptoms of cerebellar disease, and its character must be carefully investigated, as it may help to determine whether the tumour is within or external to the cerebellum. The determination of this point is of the greatest practical importance, as it is possible to remove the tumour successfully should it be extra-cerebellar. In both varieties of tumour the objective vertigo, *i.e.* the sense of movement of external objects, is from the side of the lesion to the healthy side; for example, with a tumour on the right side of the cerebellum the apparent movement of external objects is from right to left. In intra-cerebellar tumour the subjective vertigo, *i.e.* the feeling of rotation of the patient, is also from the side of the lesion to the healthy side, but in extra-cerebellar tumour it is in the opposite direction. Vertigo from this cause is associated with other symptoms, namely, headache, pain in the occipital region and back of the neck, vomiting, optic neuritis, nystagmus, ataxy of the limbs, irregular staggering gait, like that of a drunken man, paresis of the trunk muscles, so that the patient can neither bend his trunk nor raise it when lying in bed without the aid of his arms, and there is frequently convergent strabismus from sixth nerve paralysis.

*Cerebral Tumour.*—Vertigo is one of the classical symptoms of cerebral as well as of cerebellar tumour. The symptom-complex includes headache, vomiting, optic neuritis, and vertigo. It is liable to occur if the patient turns round quickly or suddenly rises from the supine to the erect position.

*Cerebral Hæmorrhage.*—Vertigo may be one of the premonitory symptoms of cerebral hæmorrhage. The cause may be determined by measuring the patient's blood-pressure; instead of being 125 millimetres of mercury it may be over 200.

### Epileptic.

A sudden attack of vertigo may constitute the aura of major epilepsy, or may be the only manifestation of a mild attack of the disease. It is usually subjective, the patient feeling that he is turning round rather than that external objects are moving. The diagnosis is usually obvious if it is followed by sudden loss of consciousness and tonic and clonic convulsions.

Even in the mild attacks there is loss of consciousness, followed by mental dulness, and this is the most distinguishing feature of vertigo due to this cause.

### Nervous Vertigo.

Giddiness is frequently one of the prominent symptoms of nervous exhaustion from overwork, mental anxiety, sexual excess, and other similar causes. It is also common in traumatic neurasthenia. It is independent of any aural disease and is never so severe as true aural vertigo. As a rule it is only felt when the patient is in the bent position, and it is most liable to occur on suddenly raising the head. It may be associated with insomnia, flatulence, nausea, and palpitation.

### Migrainous Vertigo.

Vertigo may be one of the symptoms of migraine. As a rule it is not severe, and it is associated neither with tinnitus nor deafness. It usually follows the disorders of sight and accompanies the headache which occurs in this disease. Sometimes vertigo replaces the typical attack of migraine. The association of giddiness with intense, paroxysmal, unilateral headache, transient hemianopia, flashes of light, the production of zigzag patterns and other disorders of sight, the curious spasmodic contraction and dilatation of the pupils termed hippus, and numbness of the tongue and face would point without much doubt to a diagnosis of migraine.

### Ocular Vertigo.

Vertigo is frequently associated with oculo-motor paralysis, and it is then due to the diplopia and the erroneous impressions which, in consequence, are produced as to the exact position of external objects. The giddiness is due to what is termed erroneous projection, or the false conception of the position of external objects. This form of vertigo is seldom intense, and it only occurs when the paralysed muscle is in action. This can be demonstrated by closing the paralysed eye, when the feeling of vertigo at once disappears. It may occur with paralysis of a single muscle, such as the external rectus, or may be associated with insufficiency of the internal recti muscles, a condition which is termed muscular asthenopia, which is most frequently due to myopia, but may also occur as a result of other errors of refraction or as a result of exhausting diseases such as typhoid fever and diphtheria.

When due to muscular asthenopia it usually comes on after reading. It is produced by the internal recti becoming exhausted from the prolonged strain exerted to maintain convergence of the eyes in order to focus the letters and to these muscles suddenly giving way, in consequence of which the eyeballs diverge and the letters become indistinct and a feeling of giddiness is produced. In addition to vertigo, the patient usually complains of frontal or occipital headache, nausea, and aching at the back of the eyes.

It is therefore of the greatest importance in a case of vertigo, especially if it be brought on by reading, to investigate the condition of the eyes carefully.

### Cardio-vascular.

Vertigo is a common symptom of disease of the heart and of the blood-vessels. It may be one of the earliest signs of aortic disease, the patient noticing it on suddenly rising from the supine to the erect position, as when first getting up in the morning or on raising the head after bending downwards, as in the attitude of lacing the boots. The presence of other signs and symptoms of aortic disease would point to the correct diagnosis of its cause—*i.e.* anæmia, visible and forcible pulsation of the carotids and other arteries, the characteristic splashing pulse of aortic regurgitation and the slow and continued pulse of aortic stenosis, physical signs of enlargement of the heart, the early diastolic bruit of aortic regurgitation and the systolic thrill and bruit over the second right intercostal space of aortic stenosis. Vertigo may be associated with other forms of valvular disease and also with the syncopal attacks that accompany degenerative changes in the myocardium. It is also a frequent symptom of arterio-sclerosis, and when it is associated with a slow pulse, syncopal or epileptiform attacks, it constitutes part of the syndrome termed Stokes-Adams disease. The arterio-sclerosis may or may not be associated with chronic Bright's disease. It may be detected by measuring the blood-pressure; besides which albuminuria will probably be found. Giddiness may also be produced by sudden alterations in the cerebral circulation, *e.g.* by either anæmia or hyperæmia of the brain. It is very liable to occur in women at the climateric and to be associated with other signs of vasomotor disturbances.

### Gastric and Nasal Vertigo.

This form of vertigo occurs most frequently in persons of middle age who suffer from dyspepsia. It is accompanied by neither tinnitus nor deafness, but as a rule with some of the following symptoms: epigastric pain, a feeling of distension in the upper part of the abdomen, heartburn, flatulence, palpitation, nausea, vomiting, and constipation. The attacks may come on suddenly and be followed by headache and a feeling of coldness.

It is distinguished from Menière's disease by the absence of tinnitus and deafness, and from epilepsy by there being no loss of consciousness. Gowers states that "vertigo of purely gastric origin does not constitute more than five per cent. of the cases in which definite giddiness is the prominent symptom." He believes the condition is the result of "unobtrusive" labyrinthine disease. Many cases of supposed gastric vertigo are really due to arterio-sclerosis.

Vertigo may be a symptom of nasal disease, especially of nasal obstruction from the presence of polypi. The chief evidence that it is due to such a cause is the relief which follows the cure of the nasal disease. When investigating the cause of vertigo, therefore, a careful examination of the nares should be made.

### Laryngeal, Toxic, and Nocturnal.

Laryngeal vertigo is associated with the presence of a spasmodic cough and signs of laryngeal irritation. The cough usually precedes the feeling of vertigo and appears to be the cause of it, probably by producing cerebral congestion.

When vertigo is due to sea or train sickness, or to ascents to high altitudes by mountain-climbing or in balloons, the diagnosis is obvious.

The administration of certain drugs, especially quinine and the salicylates, may give rise to well-marked vertigo. They probably act on the labyrinth through the vascular system or possibly on the auditory or cortical centres which have to do with the consciousness of auditory perceptions.

Vertigo may also be caused by the excessive use of alcohol, tobacco, or tea.

In nocturnal vertigo a feeling of giddiness or a sensation of falling from a height is experienced by the patient after retiring to bed and just before falling asleep. Gowers considers it to be a mild form of labyrinthine vertigo, which is due to a sudden spasm of the tensor tympani muscle leading to a change of pressure within the labyrinth. That it is due to contraction of an aural muscle is suggested by the fact that many affected in this way are conscious of the curious vibrating sound which is characteristic of the contraction of an intra-aural muscle.

### Endemic Paralytic Vertigo (Gerlier's Disease). (*Japan. Kubisagari.*)

This form of vertigo has been observed in Switzerland, France, and Northern Japan. The attacks of giddiness may occur once or twice a day, and they are characterised by the occurrence in addition of (*a*) weakness of the limbs and of the muscles of the neck, (*b*) drooping of the eyelids, (*c*) great mental depression, (*d*) pain in the neck and back of the head.

Attacks last from one to ten minutes, but if the patient goes on with the work he has been doing a series of attacks lasting two or three hours may be produced. The pathology of this form is not known. Both in Switzerland and in Japan it only affects country people, and especially those whose occupation brings them into close contact with cows.

